# Notes and News

**Published:** 1893-04-29

**Authors:** 


### Notes and News.

Collected and Communicated.

The Fletcher Convalescent Home at Cromer was opened on Tuesday by the Countess of Leicester and the Bishop of Norwich. We propose giving particulars of this institution later on.

Baron Nathaniel Rothschild has just presented his château at Reichenau in the Semmering to a charitable society in Vienna, for the use of patients suffering from diseases of the chest. The extensive estates and pleasure grounds are amongst the most beautiful in the Styrian Alps, and are valued at 5,000,000 florins. The Baron will formally hand over his princely gift on August 1st, and will himself provide all necessary alterations. The establishment will provide 500 beds, a number none too large for the sufferers from chest complaints. The great prevalence of phthisis in Vienna is a fact, which must strike the most casual reader of the death announcements in the Viennese papers, where the nature of the disease is, contrary to our custom, specified.

At the last Quarterly Court of the Derbyshire Royal Infirmary several new rules were added and standing rules amended Subscribers are now permitted to exchange in-patients' tickets for out-patients' tickets, three of the latter being given by the Infirmary for one of the former. All members of the weekly board are now eligible for re-election, but any Governor may nominate a new member to be voted for as before. A list of those to be voted for are to be sent to all Governors, together with the report, three days before the anniversary meeting. On the occupation of the new buildings scarlet fever and other infectious diseases will not be received into the hospital. Mr. Noble stated the welcome fact that Mr. G. Henry Strutt had forwarded a cheque for £4,000 towards the building fund, in addition to the £1,000 already given.

The international convention for taking precautions against cholera, formulated by the conference at Dresden on lines mainly suggested by the British delegate, has not yet been formally signed on behalf of Great Britain, though it is about to be so. Each country is to keep the others joining the convention informed as to outbreaks and progress of the disease, but no country is to take steps against another, and prohibition is only to be placed on such articles as can be shown capable of carrying infection.

We are glad to see that the Committee of the Bradford Infirmary call attention to the report of our Commissioner which appeared in The Hospital, December 17th, 1892. We think this example might usefully be followed by more hospitals. The Bradford Committee state "It is sometimes useful to note the impression which an institution like the infirmary produces on persons interested in similar charitable undertakings, who view it for the first time. The criticism contained in the article is candid, but its justice cannot be altogether denied, and some of the changes suggested are certainly desirable." A confident hope is expressed that it may be possible to speedily carry out the necessary alterations and improvements with the aid of the inhabitants of Bradford who must necessarily desire to place the institution on a satisfactory footing.

At the last meeting of the Metropolitan Asylums Board, a deputation of residents from Tooting presented themselves to petition against the erection of the proposed infectious hospital in their neighbourhood. The Board replied that no fresh points having been brought forward, they saw no reason for altering their choice of the site. In the meanwhile the Board is awaiting with impatience the decision of the Local Government Board in the matter. Three months have already passed in waiting, and matters are becoming so serious that the expensive expedient of erecting temporary accommodation seems likely to be necessary. Not only is scarlet fever on the increase again, but small-pox is showing alarming signs of spreading rapidly, and the less accommodation available at the outset, the greater the difficulty of eventually coping with the epidemics.

The reading of the Budget on Monday last revealed some noteworthy points of special interest. Facts are more potent than fiction, and those despondent people who are prone to regard England as increasingly enslaved to alcohol, should take courage from the words of the Chancellor of the Exchequer. He said he remembered in a previous Budget that his predecessor had commented on "a rush to alcohol"; to-day he would describe it as a stampede from alcohol. Further, he said that the discontinuance of the consumption of rum was very remarkable. In money, this decreased consumption of spirits represents about £400,000. The fact that beer remains stationary, and wine shows a slight falling off, proves that the decrease does not represent a transference of popularity to some other form of alcohol only. The gravity of the influenza epidemic is especially marked in the annals of the Treasury. The year 1892 will be ever remembered as the influenza year, during which period the increase on probate was most remarkable.

